# Effect of Various Types of Heat Processing on the Content and Retention of Fat-Soluble Vitamins and Cholesterol in Goose Breast Meat

**DOI:** 10.3390/foods14183266

**Published:** 2025-09-20

**Authors:** Zuzanna Goluch, Małgorzata Stryjecka, Gabriela Haraf, Andrzej Okruszek

**Affiliations:** 1 Department of Food Technology and Nutrition, Wroclaw University of Economics and Business, 53-345 Wroclaw, Poland; gabriela.haraf@ue.wroc.pl (G.H.); andrzej.okruszek@ue.wroc.pl (A.O.); 2 Department of Dietetics, The University College of Applied Sciences in Chełm, 22-100 Chełm, Poland; mstryjecka@panschelm.edu.pl

**Keywords:** goose, meat, heat processing, vitamin A, vitamin D, vitamin E, vitamin K, cholesterol, retention, Nutrient Reference Values

## Abstract

Background/Objectives: Heat processing techniques can alter the energy and nutritional value of meat. This study examined the effect of various types of heat processing (water bath cooking WBC, oven convection roasting OCR, grilling G, and pan frying PF) on the content and retention of vitamins A, D, E, K, and cholesterol in White Kołuda^®^ goose breast meat without or with skin (n = 36). Methods: The contents of fat-soluble vitamins and cholesterol were determined by High-Performance Liquid Chromatography (HPLC). Cooking loss (CL), retention, and the percentage coverage of the Nutrient Reference Values (NRV) for vitamins in adults by 100 g of meat were calculated. Results: The CL was higher (*p* ≤ 0.01) in goose breast meat with skin (43.2%) compared to skinless meat (37.1%). The contents of vitamins A, D, E, K, and cholesterol were also significantly greater (*p* ≤ 0.01) in meat with skin than in meat without skin. The G and PF resulted in the greatest reductions in A, D, E, and K compared with raw meat. The highest retention (>52%) was observed after WBC, whereas the lowest (<43.7%) occurred after PF, although the difference was statistically significant (*p* ≤ 0.01) only for vitamin D. While 100 g of raw goose breast meat provided the highest percentage of NRV for the analyzed components, WBC appeared to be the most favorable cooking method for consumers. Conclusions: Our research can help consumers choose goose meat as an alternative to red meat to diversify and balance their diet. WBC ensures the least loss of fat-soluble vitamins while ensuring the health safety of meat, which may be important information for consumers, the catering industry, and the poultry industry.

## 1. Introduction

In general, meat is an evolved, popular, and essential component of the human diet due to its high nutrient density relative to energy content and the bioavailability of the ingredients it contains [1]. In European countries, the average consumption of this product ranges from 40–160 g/day in children and adolescents and 75–233 g/day in adults [2]. However, consumers’ perception of meat depends on the animal species, the meat quality, and the promoted trends in meat consumption. Meat consumption trends worldwide can vary depending on the region, the animal husbandry used, traditions, religious beliefs, socioeconomic factors, nutritional deficiencies in low-income countries, and sustainable development seeking to reduce the environmental footprint of animal production [3,4,5]. In addition, recent years have highlighted the need to reduce red and highly processed meat in the human diet, the excessive consumption of which increases the risk of developing type 2 diabetes by 17%, coronary heart disease by 15%, hypertension by 14%, stroke by 12% and cancer by 11–51% [6,7]. The World Cancer Research Fund International (WCRF) recommends limiting red meat consumption to less than 500 g per week and minimizing the intake of processed meat [8], while the UK Scientific Advisory Committee on Nutrition (SACN) advises restricting red and processed meat consumption to no more than 70 g per day [2,9,10]. In this context, the European Prospective Investigation into Cancer and Nutrition (EPIC) defines white meat as poultry (including goose) and domestic rabbit, whereas some epidemiological studies also include fish in this category [11,12]. As a result, health-conscious consumers are increasingly choosing alternative sources of meat including goose meat—especially since such a dietary shift does not necessarily increase household food expenditure [13,14]. According to OECD-FAO projections, global per capita meat demand is expected to rise by 2% between the baseline period 2020–2022 and 2032 [15]. However, regional trends vary: demand is forecasted to decline in East Asia and the Pacific (accounting for 56–125% of the global decrease), while in Western Europe it may rise by 15–71% between 2020 and 2100 [16]. Currently, the world’s leading goose meat producers are China (98,53%), Taiwan (0,33%), Egypt (0.29%), Madagascar (0.29%), Myanmar (0.17%), Ukraine (0.12%), Turkiye (0.09%) while Poland, Hungary, and Belgium are the primary exporters [17]. In Poland, around 90% of goose meat comes from the White Kołuda^®^ breed; however, when combined with goat meat, it represents only 3.2% of total meat production, compared with chicken (81.6%) and turkey (15.3%). According to data from 2023, Poland produced approximately 1.11 million geese, while exports of goose meat reached close to 13.9 thousand tonnes. The majority of this output was supplied to the European Union market, including Germany, France, Italy, Austria, the Czech Republic, and the United Kingdom, as well as to Hong Kong. In terms of goose and duck meat consumption, China leads the way (76% of total meat consumption), followed by France and Myanmar. However, the highest consumption of goose and duck meat in kg/per capita/year was recorded in Taiwan, China, and Myanmar (6.12, 3.77, and 3.23, respectively) [17,18,19]. The practice of consuming raw meat remains relatively uncommon and carries the risk of microbiological hazards (*Salmonella, Campylobacter*) [20,21]. Therefore, meat is subjected to heat treatment in households, catering services, and food processing. Modern methods of thermal processing—such as vacuum cooking, infrared heating, microwave heating, ohmic heating, air frying, pressure-controlled cooking, and ultrasonic-assisted cooking—are being developed [22,23]. However, traditional heat treatments (involving water or air) such as boiling in water, steaming, baking, and frying are still the most popular techniques used by consumers and in the food service [24,25,26]. During these heat treatments (effects of temperature, heat conduction dynamics), both physical and chemical changes occur in meat, altering its composition and structure. They affect microbiological safety, the appearance, sensory quality, digestibility, energy, and nutritional value of meat [22,27]. The effects of these techniques on changes in the sensory profile of meat as a matrix, its functional properties, energy value, and specific nutrients have been investigated in several studies [18,28,29,30,31,32,33,34,35]. However, none of these studies investigated how traditional heat treatment techniques influence the levels of fat-soluble vitamins, particularly in goose breast meat, which represents the innovative aspect of the current research. Moreover, only a limited number of studies have simultaneously considered the presence of skin on the meat. Dietary guidelines and therapeutic nutrition recommendations often advise its removal before consumption, but it should be noted, however, that skin is a source of sulfur-containing amino acids, fat, collagen, elastin, minerals, and vitamins [36,37,38]. In addition, skin is one of the waste products from chicken processing, and therefore, to protect the environment, attempts are being made to use it as a raw material for the production of processed food [39] and a source of collagen or protein hydrolysates [40,41,42].

The roles played by fat-soluble vitamins in human physiology and the clinical manifestations of their deficiency or excess have been thoroughly documented in the scientific literature [43,44]. These vitamins should be supplied in the diet, preferably in the form of conventional foods rather than supplements, fortified foods, or medications. It has been shown that uncontrolled and excessive supplementation with these vitamins can not only lead to hypervitaminosis [45,46,47], but also increase the risk of exceeding the Upper Limit (UL), which for adults is set at 3000 µg for vitamin A, 100 µg for vitamin D, and 300 mg/day for vitamin E [48]. The content of fat-soluble vitamins varies depending on the type of poultry. In raw meat with skin, the vitamin A content is 50, 17, and 41 µg/100 g for duck, turkey, and chicken, respectively. The corresponding values for vitamin D are 0.7, 0.3, and 0.2 µg/100 g; for vitamin E, 0.7, 0.09, and 0.3 mg/100 g; and for vitamin K, 5.5, 0, and 1.5 µg/100 g [49].

Cholesterol performs several important physiological functions in the body. It is a structural component of cell membranes, a component of the myelin sheath of nerves and plasma lipoproteins, a precursor of steroid hormones, bile acids, and vitamin D3, and an antioxidant [50]. Drastically reducing its consumption can disrupt these processes. However, excess cholesterol in the diet, combined with saturated fatty acids, is harmful to the consumer’s health due to the risk of developing metabolic, cardiovascular, and cancer diseases [51,52]. The cholesterol content also depends on the type of poultry, and in raw meat with skin of duck, turkey, and chicken, its content is 76, 72, and 75 mg/100 g, respectively [49].

Therefore, this study aimed to: (1) determine the effect of different heat treatment techniques on the content and retention of vitamins A, D, E, K and cholesterol in goose breast meat (with or without skin); (2) evaluate the degree to which a 100 g portion of goose meat, with or without skin, may fulfill the Nutrient Reference Values (NRV) for vitamins in the adult population.

## 2. Materials and Methods

### 2.1. Materials

The present study utilized *Pectoralis Major* muscles obtained from 17-week-old White Kołuda^®^ geese, a commercial genotype developed over several decades through targeted selective breeding programs at the National Research Institute of Animal Production, Experimental Station in Kołuda Wielka, Poland [18]. All birds were raised under uniform conditions on a commercial farm and fed the same diet. Fodder contained 2.5% premixture provided to concentrate mixtures and oat grain, which consisted of fat-soluble vitamins: vitamin A (7000 IU), D_3_ (2125 IU), E (25 IU), and K (3 mg) per kilogram of feed [30,53,54].

Prior to slaughter, the geese underwent a 12-h fasting period. Slaughtering was performed at a poultry processing plant following Polish poultry industry regulations [55]. Post-evisceration, the carcasses were maintained at a controlled temperature of 2–4 °C for 24 h. Subsequently, breast muscles were excised and transported to the laboratory at Wrocław University of Economics under refrigerated conditions. Upon arrival, the muscles designated for thermal processing were sorted into groups of comparable weight. On average, muscles with skin weighed 310.9 g ± 9.6, whereas those without skin and subcutaneous fat weighed 260.6 g ± 8.6. A total of 36 breast muscle samples were analyzed, including 4 raw samples (2 with skin and 2 without) and 32 thermally processed samples (8 for each heat treatment method: 4 with skin and 4 without skin).

### 2.2. Heat Processing

The experimental procedures were adapted from an earlier study [30]. In short, goose breast muscles were subjected to heat treatments, including boiling, grilling, pan-frying, and roasting in a convection oven, until the internal muscle temperature reached 75 °C. The temperature was controlled using a manual thermometer and a thermocouple (Type T, Omega Engineering Inc., Stamford, CT, USA). Following cooling, the meat was minced and prepared for analysis. For water bath cooking (WBC), the samples were packed in plastic bags and immersed in a water bath (model SW 22, Julabo GmbH, Seelbach, Germany) at 90 °C for 30 min. Grilling (G) was carried out using an electric grill (PD 2020R, Red Fox, Warsaw, Poland) by placing the muscle between hot plates heated to 200 °C (treatment lasted 25 min). Breast muscles intended for oven convection roasting (OCR) were wrapped in aluminum foil and processed for 25 min in an oven previously heated to 200 °C (EB7551B Fusion, Amica Ltd., Wronki, Poland). Pan frying (PF) was performed by means of an electric skillet (model 48155, Unold AG, Hockenheim, Germany). The device was heated to 160 °C, and the muscle was placed on the heating surface and fried without added fat. Once the internal temperature reached 40 °C (15 min), the muscle was turned over, and cooking continued until an internal temperature of 75 °C was reached.

### 2.3. Determination of Cooking Loss (CL)

The calculation of cooking loss (CL) [35] was based on the difference between initial sample weight (Wb) before thermal treatment and final weight (Wt) measured after cooling to ambient temperature.

$$\mathrm{CL}=\frac{\mathrm{Wb}-\mathrm{Wt}}{\mathrm{Wb}}\times 100\%$$

### 2.4. Chemical Analysis

HPLC-grade chemical reagents were used in the study. All reagents used for sample preparation were from Sigma-Aldrich (St. Louis, MO, USA). Fat-soluble vitamins were determined using a method modified for the meat matrix and described by Sami et al. [56]. The modifications involved reducing the sample size, applying the Folch/Bligh-Dyer method for lipid extraction, lowering the KOH concentration during saponification, and altering the incubation conditions. To determine vitamins A, D, E, and K, exactly 1 g of meat was weighed, and lipids were extracted according to the Folch/Bligh-Dyer method (chloroform: methanol 2:1 *v*/*v*). The fat was then transferred to a clean glass test tube. 5 mL of absolute ethanol and 1 mL of 10% KOH in ethanol were added to the test tube with fat to saponify the sample. The sample was then incubated for 60 min at 60 °C in the dark, stirring occasionally. After saponification, the sample was cooled to room temperature, then 10 mL of distilled water was added and extracted 3× with 10 mL of n-hexane. The organic layers were then combined and dehydrated using anhydrous Na_2_SO_4_. Next, the sample was concentrated in a rotary vacuum evaporator (SBS-RV-5000, Steinberg Systems, Berlin, Germany) at 50 °C. The concentrated vitamin extract was diluted appropriately in methanol and filtered through a pleated filter paper into a measuring flask. The extract prepared in this way was injected into the HPLC column.

An Advanced Prominence-i high-performance liquid chromatography system (Shimadzu, Kyoto, Japan) was used for the determination of A, D, E, and K vitamins in a reversed-phase system using a Shim-pack VP-ODS column (5 μm, 4.6 × 150 mm) and a DAD detector. Spectrophotometric detection for vitamin determination was performed at the following wavelengths: A (retinol)—325 nm, D_3_ (cholecalciferol)—265 nm, E (α-tocopherol)—290 nm, and K_1_ (phylloquinone)—244 nm. A mixture of methanol and water (98:2; *v*/*v*) (Merck, Darmstadt, Germany) was used as the eluent at a flow rate of 1 mL/min. The analysis was performed at room temperature. The injection volume was 20 μL. The external standard method was used for quantitative analysis.

Cholesterol concentration was determined in two stages. The samples were saponified (first stage) according to the method of Stewart et al. [57], with minor modifications. In brief, the sample (2 g) was mixed with 4 mL of potassium hydroxide (50%) and 6 mL of ethanol (95%) until completely dissolved at 40 °C, and then heated for 10 min at 60 °C. After cooling with 5 mL of water, the unsaponifiable fraction was extracted thrice with 10 mL of n-hexane. Then, 3 mL of the extract was dried under a stream of purified nitrogen. The resulting saponified samples were analyzed by HPLC (second stage). The extracts were dissolved in 3 mL of acetonitrile and isopropanol solution (70:30, *v*/*v*), and then 1 mL was used for HPLC analysis. Advanced Prominence-i HPLC equipment was used (Shimadzu, Kyoto, Japan). The analysis was performed using a Lichrospher 5RP18 column (150 mm × 4.6 mm), and acetonitrile and isopropanol (70:30, *v*/*v*) were used as the mobile phase at a flow rate of 1 mL/min. Detection was performed at a wavelength of 210 nm. Cholesterol was identified by comparing the retention time of the sample with the standard (C8667, Sigma-Aldrich, St. Louis, MO, USA). An internal standard (6-ketocholestanol K1250, Sigma-Aldrich, St. Louis, MO, USA) was used for quantitative determination of each sample after saponification (0.504 mg per sample; final extract concentration 0.41667 mg/mL).

The validation parameters used in the analysis of vitamins and cholesterol are summarized in Table 1.

### 2.5. Determination of Retention Factor

The percentage of vitamin and cholesterol retention after heat processing (HP) was calculated by using the following equation [58]:

$$\%\;\mathrm{Retention}=\;\frac{\;nutrient\;content/100\;g\;of\;meat\;after\;HP\;}{nutrient\;content/100\;g\;of\;raw\;meat}\times \frac{meat\;weight\;\left(g\right)\;after\;HP}{meat\;weight\;\left(g\right)\;before\;HP}\times 100$$

### 2.6. Statistical Analysis

Statistical analyses were conducted using TIBCO Statistica^®^ 13.3 software. Data were tested for normality (Shapiro–Wilk) and homogeneity of variance (Levene’s test). Arithmetic means and standard errors were calculated, and differences between treatments were assessed using Tukey’s test at significance levels of *p* ≤ 0.05 and *p* ≤ 0.01. The interaction between the heat treatment method and meat type (with or without skin) was evaluated using two-way ANOVA, while one-way ANOVA was applied to compare heat treatments within each meat type. All results are presented as the average values obtained from two chemical measurements.

To explore and illustrate the associations between vitamins A, D, E, K, cholesterol levels, kind of meat, and processing methods, Principal Component Analysis (PCA) was employed. This procedure was performed using the TIBCO Statistica^®^ 13.3. Results are shown as two-dimensional biplots, PC1 vs. PC2 and PC1 vs. PC3.

## 3. Results and Discussion

### 3.1. Cooking Loss

**Figure 1:**
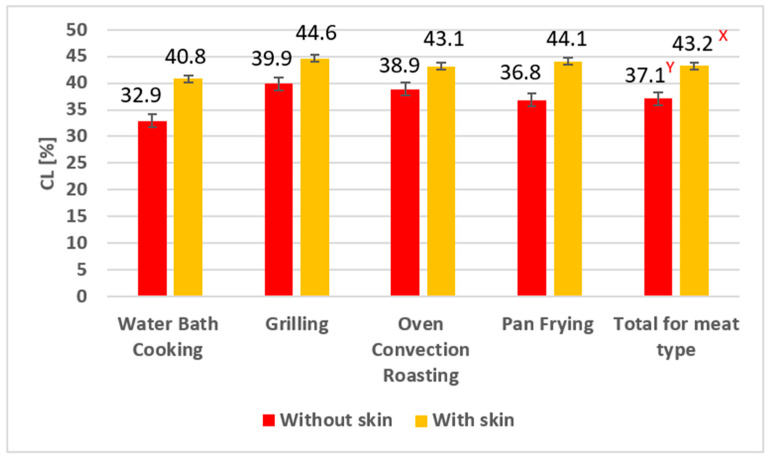
Cooking loss (CL) in goose breast meat after heat processing (means followed by different superscript letters differ significantly ^X,Y^ *p* ≤ 0.01).

The study found significantly (*p* ≤ 0.01) higher CL (Figure 1) in goose breast meat with skin (43.2%) than without skin (37.1%) for all four heat processing conditions (WBC, OCR, G, and PF). Higher CL in meat with skin may result from weight loss caused by water loss. Upon heating, the water content of meat myofibrils in the narrow channels between the fibers changes as the meat contracts within the tissue matrix, resulting in moisture loss [38]. Higher CL may also result from lipid loss under the influence of temperature [59]. Geese are waterfowl; their carcasses contain significant amounts of subcutaneous and intramuscular fat (IMF). This fat is an energy deposit during life and provides thermal insulation, especially during the early growth period [60,61]. The temperature increase during heat processing causes partial loss of epidermal lipids [39], facilitating meat juice leakage. Furthermore, Cliché et al. [41] found that processing in a hot water bath at temperatures > 64 °C causes skin protein denaturation. Together, these processes could have contributed to higher CL in meat with skin. A similar relationship was observed in other studies on White Kołuda^®^ goose breast muscles subjected to various heat treatments (grilling, sous-vide, microwave, oven convection roasting, pan frying, stewing, water bath cooking) [31,35,62].

However, our studies did not find any effect of the type of heat treatment techniques used on CL, nor any interaction between the type of meat (without skin or with skin) and processing (meat x heat processing).

### 3.2. Fat-Soluble Vitamins and Cholesterol Content in Goose Breast Meat After Heat Processing

According to various databases or food composition tables, the vitamin A content in 100 g of raw goose carcass is approximately 30–90 µg/100 g [63,64,65]. Conversely, the content of vitamin in raw skinless meat is 12–30 µg/100 g, and with skin, 17–30 µg/100 g [49,63,66,67,68]. However, these data do not apply exclusively to the breast muscles. The vitamin A content determined in our study in raw breast muscles without skin and with skin was 21.2 and 22.5 µg/100 g, respectively (Table 2). These values were higher than in raw breast muscles of Canada geese (11 µg/100 g) but lower than those found in breast muscles with bone (77.8 µg/100 g) [49,66]. However, we found a higher (*p* ≤ 0.01) content of this vitamin in goose breast meat with skin than without skin (17.7 vs. 16.7 µg/100 g), which confirms its presence in the skin tissue.

In the study, raw goose breast with skin contained 21.2 µg/100 g of vitamin A, while breast with skin contained 22.5 µg/100 g of vitamin A. Compared to literature data, these values were higher than those found in raw breast meat with skin (11 µg/100 g) from a Canada goose, but lower than those found in goose breast meat with bone (77.8 µg/100 g) [49,66].

Concerning the heat treatment of goose meat, available literature sources report data solely for roasting. According to these studies, the vitamin A content in 100 g of roasted goose ranges from 18 to 90 µg [63,65]. Furthermore, roasted goose meat without skin contains less vitamin A than meat with skin (12–15 µg/100 g vs. 17–21 µg/100 g) [49,63,66,68]. This pattern is consistent with the results of the present study, in which the corresponding values were 16.2 and 19.0 µg/100 g, respectively.

The four heat treatments we used influenced (*p* ≤ 0.01) the content of vitamin A, as indicated by the “Total” values for each treatment (Table 2). Raw and WBC goose meat contained more of this vitamin than the remaining treatments (WBC, Raw > OCR > G > PF). The reduction in vitamin A content after OCR, G, and PF can be explained by higher CL (Figure 1) and fat rendering due to heat acting on the meat and skin.

The interaction between the kind of meat (with or without skin) and heat treatment technique (meat x heat processing) was statistically significant (*p* ≤ 0.01). In skinless goose breast meat, the vitamin A content was higher in raw samples and after WBC and OCR treatments compared with G and PF (R, WBC, OCR > G, PF). Similarly, in goose breast meat with skin, the vitamin A content changed significantly (*p* ≤ 0.01) depending on the heat treatment applied, following the order WBC > R > OCR > G > PF. It may be attributed to slightly higher CL (Figure 1) observed in these treatments compared with WBC, although the differences were not statistically significant (*p* > 0.05).

Generally, vitamin D3 is found and stored in many tissues, such as adipose tissue, skeletal muscle tissue, bone, liver, intestinal mucosa, brain, and skin. Adipose tissue contains the highest concentration of vitamin D3, with skeletal muscle being the second largest storage site [69]. According to databases and food composition tables, the vitamin D content in 100 g of raw goose carcass is 0.5–1.40 µg/100 g [63,65]. In contrast, the amount of this vitamin in raw meat with skin is 1 µg/100 g, and without skin, 0.05–1 µg/100 g [65,67], although breast muscles are not specifically mentioned. In our study, the amount of this vitamin in raw breast meat without and with skin (0.33 and 0.38 µg/100 g) was similar to that described above (Table 2).

In our study, we found a higher (*p* ≤ 0.01) vitamin D content in skin-on than skin-off goose breast meat (respectively: 0.28 vs. 0.23 µg/100 g), which confirms its presence in this tissue [70]. The four heat treatments we used significantly (*p* ≤ 0.01) reduced the content of this vitamin in goose breast meat, compared to raw meat, both with and without skin (R > WBC > OCR > PF, G). The only information provided in the literature [50] concerns vitamin D levels in 100 g of roasted goose carcass, documented as 0.83 µg/100 g. This concentration is greater than the results obtained in our analyses of breast meat without (0.25 µg/100 g) and with skin (0.28 µg/100 g).

The literature provides data only on the vitamin D content in 100 g of roasted goose carcass, reported as 0.83 µg/100 g, which is higher than the values obtained in our study for breast meat without skin and with skin (0.25 and 0.28 µg/100 g, respectively).

In contrast, the American food composition database [49] reports a value of 0.1 µg/100 g for meat with skin, which is lower than our result.

According to the literature, the vitamin E content in a 100 g portion of raw goose carcass is 0.05–0.20 mg [63,65]. In our study, raw goose breast meat without skin contained less of this vitamin than with skin (respectively: 0.289 vs. 0.323 mg/100 g), which is consistent with literature data (analogously: 0.3 vs. 0.5 mg/100 g) [49]. Nowicka et al. [71] found the vitamin E content in raw White Kołuda^®^ breast meat with skin (from Małopolska, Mazovia, and Warmia-Masuria provinces) ranging from 0.31 to 0.34 mg/100 g. As shown in Table 2, the level of this vitamin was significantly higher (*p* ≤ 0.01) in skin-on goose meat compared to skin-off samples (0.253 vs. 0.220 mg/100 g), which indicates its contribution from skin tissue. Thermal treatment reduced (*p* ≤ 0.01) the vitamin E amount in goose breast meat, irrespective of skin presence, compared with raw meat (R > WBC > OCR > PF > G). According to available literature data, the vitamin E content in 100 g of roasted goose carcass is 0.33 mg/100 g [50]. In roasted meat without and with skin, the vitamin E content is reported as 0.4 and 0.5–1.74 mg/100 g, respectively [49,67], whereas the values we determined in breast meat were lower (0.202 and 0.249 mg/100 g).

The reported vitamin K content in raw goose leg muscles is 31.0 (28.2–33.1) µg/100 g [72]. However, no studies have been found that have determined its content in breast meat, which is the novelty of our research. A significantly greater (*p* ≤ 0.05) content of this vitamin (Table 2) was observed in skin-on than in skin-off meat (5.57 vs. 4.77 µg/100 g). Exposure to heat significantly diminished (*p* ≤ 0.01) the amount of vitamin K in goose breast, in samples with skin as well as skinless, compared with the uncooked control (Raw > WBC > G > OCR > PF). The vitamin K content we measured was higher than that reported for raw chicken breast without or with skin (0 µg/100 g) and for roasted chicken without or with skin (0.3 µg/100 g) [49].

According to the literature, cholesterol content in a 100 g portion of raw goose carcass ranges from 80 to 347 mg [63,64,65,73]. In raw goose meat without skin, cholesterol is reported at 84 mg/100 g, while in meat with skin it is 80 mg/100 g [49,66,67,68,74]. In our investigation, the values for raw goose breast meat were comparable but showed the opposite trend, with lower cholesterol content in skinless meat than in meat with skin (72.3 vs. 87.2 mg/100 g) (Table 2). The cholesterol level we determined for raw skinless goose breast (72.3 mg/100 g) was also lower than that reported for raw skinless breast of Canada Goose (80 mg/100 g) [49]. Nowicka et al. [71] found cholesterol contents of 52.6–62.6 mg/100 g in Kołuda goose breast meat with skin from the Małopolska, Mazovia, and Warmia–Masuria provinces. In their study, the overall difference between meat with and without skin (61.6 vs. 52.9 mg/100 g) was statistically significant (*p* ≤ 0.01), reflecting the presence of cholesterol in skin tissue.

Heat treatments significantly influenced the cholesterol content of goose breast meat relative to raw meat (R > WBC > OCR > PF > G). The reduction in this compound after roasting, frying, or grilling may be due to fat rendering and/or the formation of oxysterols [75], which requires confirmation in further studies. Only the cholesterol content in roasted goose meat has been found in the literature. A 100 g portion of roasted goose carcass contains 91–119 mg of cholesterol [63,73]. However, roasted goose meat with and without skin, on the other hand, contains 91–271 and 91–195 mg of cholesterol per 100 g, respectively [49,66,68]. The amounts of this nutrient determined in our research in goose breast meat without skin or with skin subjected to OCR were lower (45.8 and 52.3 mg/100 g) than the values quoted above.

In summary, the highest amounts of vitamins and cholesterol were found in meat treated with WBC. Among the applied techniques, roasting, grilling, and frying—classified as dry-heat treatments characterized by heat exchange through air and surface contact—resulted in the lowest detected levels of vitamins A, D, E, K, and cholesterol in the meat [23]. The reduction in vitamin and cholesterol content during oven roasting may be because during the initial roasting stage (approximately 160 °C), moisture on the meat surface evaporates rapidly, while moisture may migrate from the center of the meat to its surface. Furthermore, chemical changes occur in the meat, such as hydrolysis, oxidation, thermal decomposition, and condensation of proteins and lipids, when the temperature reaches 190 °C [27]. During grilling, meat is exposed directly to the heating element, which leads to increased water evaporation, fat liquefaction, and vitamin degradation. It is worth noting that during the OCR, G and PF, toxic compounds such as heterocyclic aromatic amines and polyaromatic hydrocarbons may be formed [54]. Therefore, they are less recommended to consumers.

### 3.3. Principal Component Analysis

A PCA was performed to visualize the associations between heat treatments, kind of meat, and the content of cholesterol and vitamins A, D, E, and K. Together, the first three principal components explained 77% of the dataset’s variance. PC1 (45%) showed high positive loadings (absolute value ≥ 0.6) for vitamins A, D, E, and K (Table 3). PC2 (17%) was positively correlated with WBC and negatively with meat type, while PC3 (12%) was positively associated with OCR and PF processing.

**Figure 2:**
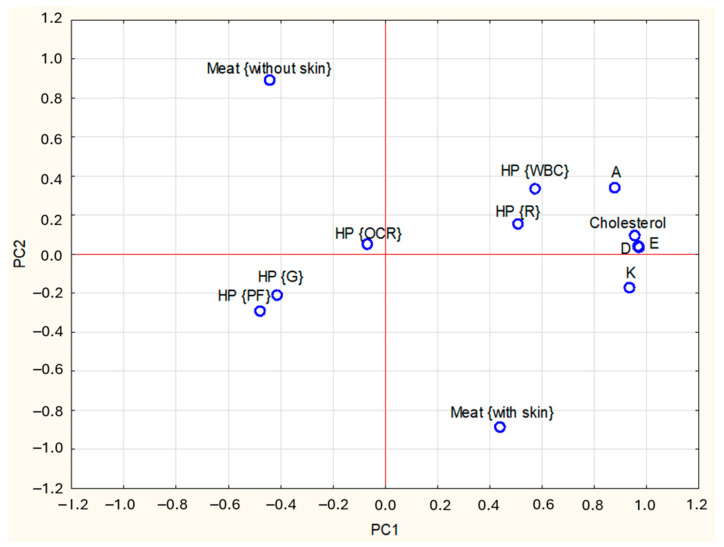
Loadings plot of the two first PCs (PC1, PC2—first and second Principal Components); HP—Heat Processing; M—type of meat; WBC—water Bath Cooking; G—Grilling; OCR—Oven Convection Roasting; PF—Pan Frying; A, D, E, K, Cholesterol—contents of particular nutrients.

**Figure 3:**
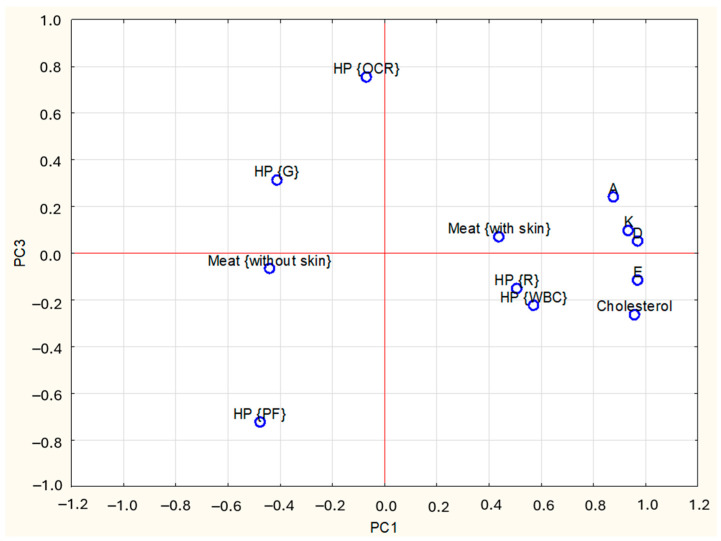
Loadings plot of the first and third PCs (PC1, PC3—first and third Principal Components); HP—Heat Processing; M—type of meat; WBC—water Bath Cooking; G—Grilling; OCR—Oven Convection Roasting; PF—Pan Frying; A, D, E, K, Cholesterol—contents of particular nutrients.

The relationship between the study results and PCs is shown graphically in Figure 2 and Figure 3. The PC1, PC2 and PC3 axes represent the principal components. Their values are a measure of variability in the data—the farther from the centre, the greater the influence of a given variable on the differences between the study groups. Values close to each other indicate a similarity of characteristics. In Figure 2 and Figure 3, the points corresponding to the vitamins and cholesterol contents are close to the WBC and raw meat, indicating significantly higher nutrient contents in these samples than in G and PF, which are on opposite sides of the graph. Figure 2 and Figure 3 also indicate that the presence of skin is positively correlated with vitamin and cholesterol contents, meaning that higher levels of these nutrients were observed in samples with skin.

### 3.4. Fat-Soluble Vitamins and Cholesterol Retention in Goose Meat After Heat Processing

Retention refers to the percentage of nutrients that remain in food after processing, assessed by the ratio of content measured before and after preparation [58]. Therefore, the retention value facilitates estimating the actual intake of a given nutrient from processed meat. This value can also help identify the processing method characterized by the highest degree of retention of individual nutrients. In the present study, retention was calculated for the analyzed components, expressing the proportion of nutrients remaining in heat-treated goose meat relative to their initial amounts (raw meat), while accounting for weight changes before and after processing. This approach provides a more accurate estimate of nutrient preservation. There is limited literature on the stability of fat-soluble vitamins—sensitive to pH, oxygen, and elevated temperatures—in goose meat subjected to various heat treatments. Available studies [58,76] indicate that their reduction during cooking results mainly from three interrelated processes: (1) direct thermal degradation at high temperatures, (2) oxidative degradation, and (3) physical losses through leaching into cooking liquids or migration with rendered fat, as observed during boiling and frying.

Unlike the heat treatment technique, the presence or absence of skin did not significantly affect vitamin A retention (Table 4). In the case of meat with skin, the retention of this vitamin ranged from 32.1% for PF meat to 66.8% for WBC meat. Ignoring the type of meat and taking into account only the kind of heat treatment (“Total for HP” value in Table 4), significantly (*p* ≤ 0.01; *p* ≤ 0.05) higher retention of this vitamin was found in WBC goose breast meat (65.3%), compared to OCR, G, and PF (46.7; 38.0 and 32.4%, respectively).

The interaction between meat type and heat treatment was statistically significant. In skinless meat, vitamin A retention was higher after WBC and OCR compared to G and PF (63.5 and 51.4% vs. 37.5 and 32.7%, *p* ≤ 0.01). For meat with skin, the highest retention was again observed in WBC (WBC > OCR, G > PF). Overall, cooked meat retained the most vitamin A, while fried meat retained the least. No comparable studies on geese were found, but similar trends were reported in beef, pork, chicken, and lamb, with vitamin A retention ranging from 15.4% to 91% depending on processing method [77,78].

Vitamin D retention (Table 4) in goose breast meat ranged from 32.3% (PF without skin) to 56.4% (WBC with skin). The higher (*p* ≤ 0.01) vitamin D retention in goose breast meat with skin than without skin (43.0 vs. 38.8%) may be due to the structure of the skin. Although no studies were found on the chemical composition of goose skin, it is worth considering the composition of chicken skin. This skin comprises a thick dermis (inner layer) and a thin epidermis (outer layer) with a lipid content of approximately 45%. Chicken epidermal lipids include wax diesters (34%), triglycerides (32%), sterols (11%), phospholipids (11%), and other smaller lipids. Epidermal lipids function as a protective barrier [39]; therefore, in our opinion, vitamin D losses are lower. Additionally, vitamin D is present in the skin as 7-dehydrocholesterol. Among the applied heat treatments, WBC resulted in markedly greater retention relative to OCR, PF, and G, regardless of whether the meat was with or without skin (WBC > OCR > PF, G). Among heat treatments, WBC preserved the most vitamin D, followed by OCR, PF, and G. No comparable data for geese were found, although studies in beef report higher retention (60–90%) after various cooking methods, attributed to its lower fat content and reduced vitamin losses [58].

Vitamin E retention in goose breast meat ranged from 37.0% (G without skin) to 58.8% (WBC with skin) (Table 4). The presence of skin had no significant effect, but retention was markedly higher after WBC compared to OCR, PF, and G (58.1 vs. 42.8, 39.8, and 37.6%, *p* ≤ 0.01). No comparative data are currently available regarding the influence of diverse culinary processing methods on the retention of this vitamin in goose meat. It has been shown [79] that grilling meat (beef, pork, and veal) reduces vitamin E content by 11–21.8%, while boiling, frying, and baking rabbit meat reduces it by 39%, 12%, and 14%, respectively [80]. According to Lešková et al. [58], vitamin E retention in different types of meat subjected to various heat treatments ranges from 44% to 95%, which is higher than the values obtained in our study for goose breast meat.

The calculated vitamin K retention (Table 4) in goose breast meat ranged from 42.3% (G without skin) to 61.2% (WBC with skin). No significant effect of muscle type (without or with skin) on vitamin K retention was found, but significantly (*p* ≤ 0.01) higher retention was observed in WBC meat than after other heat treatments. In muscles with skin, vitamin K retention was higher in WBC (61.2%) than in PF (43.7%) and OCR (46.2%). No studies were found on the stability of vitamin K in cooked muscle tissue of meat.

Cholesterol retention (Table 4) in meat ranged from 31.5% (G without skin) to 59.5% (WBC with skin). Cholesterol retention in cooked meat (regardless of whether with or without skin) was higher (*p* ≤ 0.01) than after other treatments (WBC > OCR, PF, G). However, there is a visible trend of greater retention of this nutrient in meat with skin than without skin. It is due to the presence of cholesterol as a sterol compound found in the skin [39]. There are no data in the literature on the cholesterol retention in goose meat after cooking, grilling, baking, and frying. However, after applying other processing methods (sous vide, microwave, and stewing), significantly higher retention was found in White Kołuda^®^ goose breast meat with skin (99.2%) than without skin (93.3%) [28]. It was also shown that meat after sous vide processing had the highest retention of this component (106.0%), and stewed meat had the lowest (86.2%).

In summary, it can be concluded that the highest retention (lowest losses) in all analyzed components was observed in WBC goose breast meat, and the lowest in G and PF meat.

### 3.5. Goose Meat and Nutrient Reference Values (NRV) for Adults

According to existing legal requirements [81,82], manufacturers are obliged to indicate on product labels the energy value and nutrient content, expressed per 100 g or 100 mL. The label should also include the Nutrient Reference Value (NRV), which is based on scientific knowledge about the daily requirement of energy or nutrients an adult needs to maintain good health [82]. This information enables consumers to make informed nutritional choices that support a diversified and balanced diet. According to Annex XIII to Regulation (EU) No 1169/2011 of the European Parliament and of the Council of 25 October 2011 on the provision of food information to consumers, the NRV-R for vitamins A, D and E and K is, respectively, 800, 5, 900 and 60 μg/100 g or 100 mL of product [81].

In goose breast meat, the presence of skin significantly (*p* ≤ 0.01) increased the share of NRV covered for all fat-soluble vitamins (Table 5). On average, meat with skin provided higher values than skinless meat. Heat treatment significantly reduced NRVs (*p* ≤ 0.01 or *p* ≤ 0.05), with retention following the order: raw > WBC > OCR > PF > G. This pattern was consistent for both meat with and without skin. In conclusion, raw goose breast meat provides the highest NRV contribution but is unsuitable for consumption due to microbiological and sensory concerns. Among heat treatments, WBC was most effective in preserving vitamins A, D, E, and K.

According to the Regulation [81], a food can be labelled as a “source” of a nutrient if it provides at least 15% of the NRV, and a “high source” if it provides 30% of the NRV for adults per 100 g. Our research shows that 100 g of goose meat does not provide fat-soluble vitamins in the amounts indicated above, so it cannot be called a “source” or “high source” but can still be part of a varied and balanced diet.

It is worth noting that NRVs for adults are average values and do not take into account age and gender groups, which in turn are included in the nutrient intake standards in different countries at different levels (e.g., Adequate Intake—AI, Estimated Average Requirement—EAR, Population Reference Intake—PRI, Recommended Intake—RI, Recommended Dietary Allowance—RDA). Table 6 shows the recommended intake of individual fat-soluble vitamins by various national and international institutions, i.e., Nutrition Societies in Germany and Austria and Switzerland [83], The Health Council of the Netherland [84], Nordic Nutrition Recommendations [85], European Food Safety Authority [86], French Agency for Food [87] and National Institute of Public Health-National Institute of Hygiene (Poland) [88].

It is also important to note that consuming 100 g of goose breast meat will certainly not exceed the Upper Limit for vitamins A, D, and E, which are 3000, 100, and 300 µg/day for an adult. No UL has been established for vitamin K [48].

No NRV has been established for cholesterol because endogenous synthesis is sufficient to meet the body’s cholesterol needs. Until recently, dietary prevention has been recommended to limit cholesterol intake to below 300 mg/day [89]. Considering these recommendations, 100 g of goose meat provides from 41.3 to 87.2 mg of cholesterol, lower than the recommended <300 mg/day (13.8–29.1%). However, the current literature does not support the view that dietary cholesterol increases the risk of heart disease in healthy individuals [90]. The 2020–2025 Dietary Guidelines for Americans recommend keeping dietary cholesterol intake as low as possible without compromising the diet’s nutritional value [91].

To summarize our study, we believe that WBC appears to be the optimal heat treatment method for preserving fat-soluble vitamins and cholesterol in goose breast meat. This treatment was characterized by the highest retention of vitamins A, D, E, and K (in skin-on and skin-off meat) and lower thermal losses. In previous sensory studies, panelists considered WBC meat highly desirable in terms of overall palatability. This supports the idea that WBC meat is the optimal heat treatment method [31]. According to literature data, WBC treatment also carries the lowest risk of producing harmful compounds. Compounds such as heterocyclic aromatic amines (HAA), polycyclic aromatic hydrocarbons (PAH), N-nitrosamines (NA), acrylamide, and oxysterols can be formed during home roasting, grilling, and frying, as well as during industrial meat processing [92,93,94]. These toxic compounds raise concerns among meat consumers about their health safety [95].

### 3.6. Limitations

Our study was limited by the use of a single, domesticated goose genotype (White Kołuda), a single muscle type (breast), and a limited sample size for each heat treatment technique. The use of only common heat treatment methods may also be a limitation; therefore, it would be advisable to include modern processing methods such as sous vide, air frying, ohmic heating, microwave, radio frequency, and infrared heating in future studies. The lack of literature on fat-soluble vitamin content in goose meat makes comparing our results with other research difficult.

## 4. Conclusions

This study demonstrated significantly higher levels of vitamins A, D, E, K, and cholesterol in skin-on meat compared to skinless meat, as the skin helps limit vitamin losses during processing. However, significantly higher retention in skin-on meat was observed only for vitamin D. All heat treatment methods led to a significant reduction in nutrient content compared to raw meat, with grilling and pan-frying causing the greatest losses. Consequently, nutrient retention varied significantly between techniques, with the highest values (above 52%) after WBC processing and the lowest (below 43.7%) after pan-frying. A 100 g portion of WBC-treated goose breast meat provides the greatest contribution to the NRV for vitamins A, D, E, and K, making this method the most beneficial for consumers. Since intake standards for these vitamins differ by country and gender, the percentage of NRV coverage should be considered in relation to the consumer’s profile and region. Knowledge of the content of fat-soluble vitamins and cholesterol in goose breast meat, as well as the effects of different heat treatments, may influence consumer choice of goose meat as a healthier alternative to red meat, and guide both consumers and foodservice professionals in selecting cooking methods that minimize nutrient losses.

## Figures and Tables

**Table 1:** Validation parameters used in the analysis.

Nutrient	LOD [μg/mL]	LOQ [μg/mL]	Precision [%]	Recovery [%]	Uncertainty [%]
A	0.44	1.35	0.37	99.91	0.09
D_3_	0.31	0.94	2.04	101.0	0.01
E	0.94	2.86	0.60	99.98	1.98
K_1_	0.29	0.88	0.27	99.97	0.02
Cholesterol	0.72	2.17	0.28	100.4	0.23

LOD—limit of detection, LOQ—limit of quantification.

**Table 2:** Fat-soluble vitamins and cholesterol content (per 100 g FM) in White Kołuda^®^ goose breast, raw and after heat processing (HP).

Item	Meat Type	Raw	Heat Processing				Total for Meat Type	SEM	Level of Significance		
			Water Bath Cooking (WBC)	Grilled (G)	Oven Convection Roasting (OCR)	Pan Fried (PF)			Meat Type (M)	Heat Processing (HP)	M × HP
Vitamin A (µg)	without skin	21.2 ^A^	20.7 ^A^	13.6 ^B^	19.0 ^A^	11.3 ^B^	16.7 ^Y^	0.98	0.001	0.001	0.001
	with skin	22.5 ^B^	24.6 ^A^	15.2 ^D^	16.2 ^C^	12.6 ^E^	17.7 ^X^	1.11			
	Total for HP	21.9 ^A^	22.7 ^A^	14.4 ^C^	17.6 ^B^	12.0 ^D^	17.2	0.74			
	SEM	0.38	0.75	0.60	0.54	0.23					
Vitamin D (µg)	without skin	0.33 ^A^	0.27 ^B^	0.17 ^D^	0.25 ^C^	0.18 ^D^	0.23 ^Y^	0.013	0.001	0.001	0.004
	with skin	0.38 ^A^	0.34 ^B^	0.22 ^D^	0.28 ^C^	0.28 ^D^	0.28 ^X^	0.013			
	Total for HP	0.35 ^A^	0.30 ^B^	0.19 ^D^	0.26 ^C^	0.20 ^D^	0.25	0.010			
	SEM	0.013	0.012	0.010	0.006	0.009					
Vitamin E (µg)	without skin	289.5 ^A^	260.4 ^B^	187.6 ^E^	202.8 ^C^	196.6 ^D^	220.5 ^Y^	8.82	0.001	0.001	0.001
	with skin	323.7 ^A^	303.6 ^B^	210.5 ^E^	249.9 ^C^	214.8 ^D^	253.5 ^X^	10.4			
	Total for HP	306.6 ^A^	282.0 ^B^	199.1 ^E^	226.4 ^C^	205.7 ^D^	337.0	7.29			
	SEM	9.92	8.21	4.36	8.91	3.46					
Vitamin K (µg)	without skin	5.61 ^A^	5.18 ^B^	4.30 ^D^	4.98 ^C^	4.17 ^E^	4.77 ^y^	0.12	0.028	0.013	0.002
	with skin	6.76 ^A^	6.34 ^B^	5.53 ^C^	5.00 ^D^	4.82 ^E^	5.57 ^x^	0.17			
	Total for HP	6.19 ^A^	5.76 ^B^	4.92 ^D^	4.99 ^C^	4.50 ^E^	5.17	0.12			
	SEM	0.33	0.22	0.23	0.02	0.12					
Cholesterol (mg)	without skin	72.3 ^A^	69.4 ^B^	41.3 ^E^	45.8 ^C^	45.3 ^D^	52.9 ^Y^	3.03	0.001	0.001	0.001
	with skin	87.2 ^A^	79.4 ^B^	50.2 ^E^	52.3 ^C^	51.5 ^D^	61.6 ^X^	3.55			
	Total for HP	79.8 ^A^	74.4 ^B^	45.8 ^E^	49.0 ^C^	48.4 ^D^	57.2	2.41			
	SEM	4.30	1.90	1.69	1.23	1.17					

Values in the same row marked with different superscript letters differ significantly; ^A,B,C,D,E^ *p* ≤ 0.01; Values in the same column with different superscripts differ significantly; ^X,Y^ *p* ≤ 0.01, ^x,y^ *p* ≤ 0.05; SEM—Standard Error of a Mean. The experimental material consisted of 12 raw breast muscles with skin and 12 without skin, and for each thermal treatment, 6 with skin and 6 without skin.

**Table 3:** Loadings for the first three PCs.

	Items	PC1 ^1^	PC2 ^1^	PC3 ^1^
Fat-soluble vitamins contents:	A	0.88	0.37	0.24
	D	0.97	0.07	0.05
	E	0.96	0.05	−0.11
	K	0.94	−0.04	0.10
	Cholesterol	0.96	0.09	−0.26
Type of meat (M):	M {without skin}	−0.47	0.87	−0.07
	M {with skin}	0.47	−0.87	0.07
Kind of heat processing (HP):	HP {raw}	0.49	0.16	−0.15
	HP {WBC}	0.54	0.33	−0.23
	HP {G}	−0.39	−0.22	0.31
	HP {OCR}	−0.00	0.10	0.75
	HP {PF}	−0.51	−0.33	0.73

^1^ PC1, PC2, PC3—first, second and third Principal Components; WBC—water Bath Cooking; G—Grilling; OCR—Oven Convection Roasting; PF—Pan Frying.

**Table 4:** Retention (%) coefficients of fat-soluble vitamins and cholesterol in heat-processed White Kołuda^®^ goose breast.

Item	Meat Type	Raw	Heat Processing				Total for Meat Type	SEM	Level of Significance		
			Water Bath Cooking (WBC)	Grilled (G)	Oven Convection Roasting (OCR)	Pan Fried (PF)			Meat Type (M)	Heat Processing (HP)	M × HP
Vitamin A	without skin	-	63.7 ^A^	37.5 ^Bb^	51.4 ^Aa^	32.7 ^B^	46.3	3.50	0.415	0.001	0.079
	with skin	-	66.8 ^A^	38.6 ^B^	42.1 ^B^	32.1 ^C^	44.9	3.44			
	Total for HP	-	65.3 ^A^	38.0 ^Ca^	46.7 ^B^	32.4 ^Cb^	45.6	2.42			
	SEM	-	2.89	1.60	1.78	0.94					
Vitamin D	without skin	-	52.4 ^A^	28.8 ^B^	41.8 ^A^	32.3 ^B^	38.8 ^Y^	2.74	0.005	0.001	0.697
	with skin	-	56.4 ^A^	34.7 ^C^	44.8 ^B^	36.1 ^C^	43.0 ^X^	2.35			
	Total for HP	-	54.4 ^A^	31.8 ^C^	43.3 ^B^	34.2 ^C^	40.9	1.81			
	SEM	-	2.94	1.54	1.06	1.00					
Vitamin E	without skin	-	57.4 ^A^	37.0 ^B^	39.3 ^B^	40.5 ^B^	43.5	2.56	0.158	0.001	0.271
	with skin	-	58.8 ^A^	38.3 ^Bb^	46.4 ^Ba^	39.1 ^Bb^	45.7	2.25			
	Total for HP	-	58.1 ^A^	37.6 ^B^	42.8 ^B^	39.8 ^B^	44.6	1.69			
	SEM	-	2.95	1.30	1.48	0.96					
Vitamin K	without skin	-	57.0	42.3	48.1	43.8	47.5	2.36	0.211	0.001	0.457
	with skin	-	61.2 ^Aa^	50.2	46.2 ^b^	43.7 ^B^	50.3	2.17			
	Total for HP	-	59.1 ^Aa^	46.2 ^B^	47.2 ^b^	43.2 ^B^	48.9	1.60			
	SEM	-	3.67	2.64	1.51	1.22					
Cholesterol	without skin	-	59.3 ^A^	31.5 ^B^	34.3 ^B^	36.0 ^B^	40.2	3.36	0.220	0.001	0.811
	with skin	-	59.5 ^A^	35.3 ^B^	37.5 ^B^	36.2 ^B^	42.1	2.80			
	Total for HP	-	59.4 ^A^	33.4 ^B^	35.9 ^B^	36.1 ^B^	41.2	2.16			
	SEM	-	3.70	1.72	1.14	0.95					

SEM—Standard Error of a Mean; Means within a row followed by different superscript letters differ significantly; ^A,B,C^ *p* ≤ 0.01; ^a,b^ *p* ≤ 0.05; Means within a column followed by different superscript letters differ significantly; ^X,Y^ *p* ≤ 0.01; n = 6 breast muscles with skin and n = 6 without skin for each kind of heat processing.

**Table 5:** Realisation (%) of Nutrient Reference Values (NRV) for fat-soluble vitamins through intake of 100 g of White Kołudzka^®^ goose breast, raw or thermally processed (HP).

Item	NRV ^1^ (μg)	Meat Type	Raw	Heat Processing				Total for Meat Type	SEM	Level of Significance		
				Water Bath Cooking (WBC)	Grilled (G)	Oven Convection Roasting (OCR)	Pan Fried (PF)			Meat Type (M)	Heat Processing (HP)	M × HP
Vitamin A	800	without skin	2.65 ^A^	2.59 ^A^	1.70 ^B^	2.37 ^A^	1.42 ^B^	2.09 ^Y^	0.122	0.006	0.001	0.001
		with skin	2.82 ^B^	3.08 ^A^	1.90 ^D^	2.02 ^C^	1.57 ^E^	2.22 ^X^	0.139			
		Total for HP	2.79 ^A^	2.83 ^A^	1.80 ^C^	2.20 ^B^	1.49 ^D^	2.15	0.092			
		SEM	0.048	0.094	0.075	0.067	0.029					
Vitamin D	5	without skin	6.63 ^A^	5.47 ^B^	3.37 ^Db^	4.97 ^C^	3.62 ^Da^	4.61 ^Y^	0.268	0.001	0.001	0.005
		with skin	7.50 ^Aa^	6.70 ^Ab^	4.42 ^C^	5.55 ^B^	4.57 ^C^	5.55 ^X^	0.270			
		Total for HP	7.07 ^A^	6.08 ^B^	3.89 ^Db^	5.26 ^C^	4.09 ^Da^	5.08	0.204			
		SEM	0.260	0.240	0.205	0.115	0.187					
Vitamin E	9000	without skin	3.22 ^A^	2.89 ^B^	2.08 ^E^	2.25 ^C^	2.18 ^D^	2.45 ^Y^	0.098	0.001	0.001	0.001
		with skin	3.60 ^A^	3.37 ^B^	2.34 ^E^	2.78 ^C^	2.39 ^D^	2.82 ^X^	0.116			
		Total for HP	3.41 ^A^	3.13 ^B^	2.21 ^E^	2.52 ^C^	2.29 ^D^	2.63	0.081			
		SEM	0.110	0.091	0.048	0.099	0.038					
Vitamin K	60	without skin	5.36 ^A^	4.82 ^B^	3.47 ^E^	3.76 ^C^	3.64 ^D^	4.08 ^Y^	0.163	0.001	0.001	0.001
		with skin	5.99 ^A^	5.62 ^B^	3.90 ^E^	4.63 ^C^	3.98 ^D^	4.69 ^X^	0.193			
		Total for HP	5.68 ^A^	5.22 ^B^	3.69 ^E^	4.19 ^C^	3.81 ^D^	4.39	0.135			
		SEM	0.184	0.152	0.081	0.165	0.064					

^1^ NRV—Nutrient Reference Values; Means within a row followed by different superscript letters differ significantly; ^A,B,C,D,E^ *p* ≤ 0.01; ^a,b^ *p* ≤ 0.05; Means within a column followed by different superscript letters differ significantly; ^X,Y^ *p* ≤ 0.01.

**Table 6:** Recommendations for the adults’ intake of fat-soluble vitamins set by various national and international institutions.

Institution	A [μg of Retinol Equivalent/Day]		D [μg/Day]		E [mg of α-Tocopherol Equivalent/Day]		K [μg/Day]	
	♀	♂	♀	♂	♀	♂	♀	♂
Nutrition Societies in Germany and Austria and Switzerland [83]	700 ^1)^	800–850 ^1)^	20	20	11–12	12–15 ^2)^	60–65 ^2)^	70–80 ^2)^
The Health Council of the Netherland [84]	600	800	20	20	11	13 ^2)^	70 ^2)^	70 ^2)^
Nordic Nutrition Recommendations [85]	650–700 ^4)^	750–900 ^4)^	10–20 ^3)^	10–20 ^3)^	9–10 ^3)^	11 ^3)^	60–65 ^2)^	70–75 ^2)^
European Food Safety Authority [86]	650 ^1)^	750 ^1)^	15 ^2)^	15 ^2)^	11	13 ^2)^	70 ^2)^	70 ^2)^
French Agency for Food [87]	650 ^1)^	750 ^1)^	15 ^2)^	15 ^2)^	12 ^2)^	12 ^2)^	79 ^2)^	79 ^2)^
National Institute of Public Health- National Institute of Hygiene (Poland) [88]	700 ^4)^	900 ^4)^	15	15	8 ^2)^	10 ^2)^	55 ^2)^	65 ^2)^

♀—female; ♂—male; ^1)^ PRI—Population Reference Intake; ^2)^ AI—Adequate Intake; ^3)^ RI—Reference Intake; ^4)^ RDA—Recommended Dietary Allowance.

## Data Availability

The data presented in this study are available on request from the corresponding author.

## Abbreviations

The following abbreviations are used in this manuscript:

AI	Adequate daily Intake
CL	Cooking loss
EFSA	European Food Safety Authority
HAA	Heterocyclic Aromatic Amines
HP	Heat processing
FAO	Food and Agriculture Organization of the United Nations
G	Grilling
LOD	Limit of detection
LOQ	Limit of quantification
NRV	Nutrient Reference Values
OCR	Oven Convection Roasting
OECD	Organization for Economic Co-operation and Development
PAH	Polycyclic Aromatic Hydrocarbons
PCA	Principal Component Analysis
PF	Pan Frying
UL	Upper Level
USDA	United States Department of Agriculture
WBC	Water Bath Cooking
WHO	World Health Organization
